# Plant Interactions with Changes in Coverage of Biological Soil Crusts and Water Regime in Mu Us Sandland, China

**DOI:** 10.1371/journal.pone.0087713

**Published:** 2014-01-31

**Authors:** Shuqin Gao, Xu Pan, Qingguo Cui, Yukun Hu, Xuehua Ye, Ming Dong

**Affiliations:** 1 State Key Laboratory of Vegetation and Environmental Change, Institute of Botany, Chinese Academy of Sciences, Beijing, China; 2 Key Laboratory of Hangzhou City for Ecosystem Protection and Restoration, Hangzhou Normal University, Hangzhou, China; 3 University of Chinese Academy of Sciences, Beijing, China; USDA-ARS, United States of America

## Abstract

Plant interactions greatly affect plant community structure. Dryland ecosystems are characterized by low amounts of unpredictable precipitation as well as by often having biological soil crusts (BSCs) on the soil surface. In dryland plant communities, plants interact mostly as they compete for water resources, and the direction and intensity of plant interaction varies as a function of the temporal fluctuation in water availability. Since BSCs influence water redistribution to some extent, a greenhouse experiment was conducted to test the hypothesis that the intensity and direction of plant interactions in a dryland plant community can be modified by BSCs. In the experiment, 14 combinations of four plant species (*Artemisia ordosica*, *Artemisia sphaerocephala*, *Chloris virgata* and *Setaria viridis*) were subjected to three levels of coverage of BSCs and three levels of water supply. The results show that: 1) BSCs affected plant interaction intensity for the four plant species: a 100% coverage of BSCs significantly reduced the intensity of competition between neighboring plants, while it was highest with a 50% coverage of BSCs in combination with the target species of *A. sphaerocephala* and *C. virgata*; 2) effects of the coverage of BSCs on plant interactions were modified by water regime when the target species were *C. virgata* and *S. viridis*; 3) plant interactions were species-specific. In conclusion, the percent coverage of BSCs affected plant interactions, and the effects were species-specific and could be modified by water regimes. Further studies should focus on effects of the coverage of BSCs on plant-soil hydrological processes.

## Introduction

Plant interactions can greatly affect plant distribution, dynamics and diversity, thus affect plant community structure and function [1]–[3]. Among plant interactions, competition (negative effects) has long been thought to be a mechanism to promote community stability in a variable environment [4], [5]; on the other hand, facilitation (positive effects) has been considered to be a positive mechanism for community succession [6], [7]. Such different plant interactions may operate simultaneously among different species [8], while shaping a plant community [9] or even an entire ecosystem [1], [2]. Plant interactions may vary, in terms of direction and intensity along environmental gradients [3], especially as mediated by resource availability [10]. For example, in some cases the relationship between two species shifts from competition to facilitation in response to increasingly severe environmental conditions [8], [11]. With increasing environmental stress, competition in some plant communities decreased while facilitation became more important [12]. In one study in a semiarid plant community, the direction and intensity of plant interactions varied as a function of intra- and inter-annual water availability [13].

Dryland ecosystems are characterized by low amounts of unpredictable precipitation [14]–[17], such as occurs in the arid and semiarid areas of China. Such arid and semiarid ecosystem often possess vegetation with sparse aboveground organs but dense below-ground organs, as well as often being associated with biological soil crusts (BSCs) on the soil surface [18], [19]. In such ecosystems, plant interactions occur mostly belowground. For instance, they often compete for soil water resources [10] which are redistributed to some extent by BSCs [20]–[23]. In this study, we examine how plant interactions respond to the percent coverage of BSCs in three different water regimes in dryland plant communities. Since BSCs can influence water redistribution in dryland plant communities and water availability may affect plant interactions, we hypothesize that in dryland plant communities, 1) the intensity and direction of plant interactions are influenced by percent coverage of BSCs and 2) the effects of percent coverage of BSCs on plant interactions are modified by water regime.

To test these hypotheses, a greenhouse experiment was conducted in semiarid North China, in which 14 species combinations of four dryland plant species were subjected to three levels of percent BSCs coverage and three levels of water supply. A relative interaction index (RII) [24] was employed as a measure of the intensity and direction of plant interaction.

## Materials and Methods

### Study site

We conducted a greenhouse experiment at the Ordos Sandland Ecological Station (OSES) (39°02′N, 109°21′E), Institute of Botany, Chinese Academy of Sciences, located in the Mu Us Sandland, China. This semiarid area has a typical continental climate with a mean annual precipitation of ca. 300 mm, occurring mostly (60–70%) between July and September. The annual mean temperature is 6.2–8.5°C, with monthly means of 22°C in July and −1°C in January [25]–[27].

### Study species

*Artemisia ordosica* Krasch. (Asteraceae) is a dominant shrub species in the fixed and semi-fixed sand dunes with plumose, linearly lobate leaves. Its branch roots are mainly distributed in the upper 30 cm of the sand soil profile, while its primary roots may reach 1–3 m deep [28]; *Artemisia sphaerocephala* Krasch. (Asteraceae) is one of the most important pioneer plants on the moving and semi-fixed sand dunes, with strong resistance to drought, cold and saline-alkaline soil conditions [29]. *Chloris virgata* Swartz (Poaceae) and *Setaria viridis* (L.) Beauv. (Poaceae) are annual grass species, widely distributed in roadsides, abandoned land and sandy soils.

Seeds for the experiment were collected near the OSES as they matured in September 2007 for *C. virgata* and *S. viridis* and in November 2007 for *A. ordosica* and *A. sphaerocephala*.

### Experimental design and measurements

A total of 882 containers (15 cm diameter and 13 cm height) were prepared; each was filled with 1,100 ml sand which had been collected near the OSES, and sieved to remove the soil's seed bank. We planted 5–10 seeds of the four species in these pots on June 12, 2008. Fifteen days later, we selected similar sized seedlings for our experiment and removed any large or small plants. Fourteen species combinations were set up as follows: four had a single seedling of one of the four test species in one pot, four had two seedlings of one of the test species in one pot, and the other six combinations had two seedlings, with one seedling of each of two different species in one pot. For each of these 14 species combinations, three levels of water supply and three levels of BSCs coverage were set up for a total of 126 treatment types, each with seven replicates or a grand total of 882 test pots. The three water levels were 80 ml, 120 ml and 160 ml every 3 days, simulating precipitation of 200, 300, and 400 mm in a growing season, respectively. Three levels of percent BSCs coverage were 0%, 50% and 100%. BSCs were collected from the *A. ordosica* communities near the station, for their uniform thickness (about 0.8 cm) and simple species composition (just one species *Bryum argenteum*). In each BSCs treatment by adding a soil crust, a 0.8 cm deep layer of crust was placed over sand in each pot. The experiment was conducted from June 28 to September 28, 2008. On 26 July and 20 August, 20 ml nutrient solution (Peters professional: 20% N, 20% P_2_O_5_ and 20%K_2_O, the Scotts Company, Ohio, USA) were supplied to each pot. Mean air temperature was 24.1°C and mean air humidity was 57.3% in greenhouse (Thermo Datalogger, Campbell Inc., Logan, UT, USA) during the experimental period.

At the end of the experiment, all plants were harvested, dried at 75°C for 48 h, and then weighed. Using biomass data, we calculated an RII as a measure of plant interaction intensity and direction [24]. RII has strong mathematical and statistical properties, which overcome problems experienced with other frequently used indices [30], Equation (1) was used:
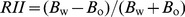
(1)where *B*_w_ is the biomass of target plant growing with a neighbor, noting that the neighbor plants may be either the same or a different species in our experiment, and the mean value is used while the neighbor plants belong to the same species; *B*_o_ is the biomass of a target plant growing in absence of inter- or intra-specific interactions, that is, the biomass of a single seedling planted by itself in our experiment.

The target plant is said to have experienced a (positive) facilitative effect from a neighbor plant if RII>0 and a (negative) competitive effect if RII<0. A greater absolute value of RII indicates a greater intensity of plant interaction.

### Statistical analyses

Three-way ANOVA was used to test effects of BSCs coverage, water regime and neighboring plants on RII of each target plant species. Two-way ANOVA was used to analyze the effects of BSCs coverage and water regime on RII of each target plant species. The effects of percent BSCs coverage on RII, the effects of neighboring plants on RII, and the effects of percent BSCs coverage on biomass of each target plant species were analyzed separately using one-way ANOVA. Data were transformed to meet normality and homogeneity of ANOVA, if necessary. All statistical analyses were performed using SPSS 17.0 (SPSS, Chicago, IL, USA).

## Results

### Effects of BSCs coverage on plant interactions

**Figure 1:**
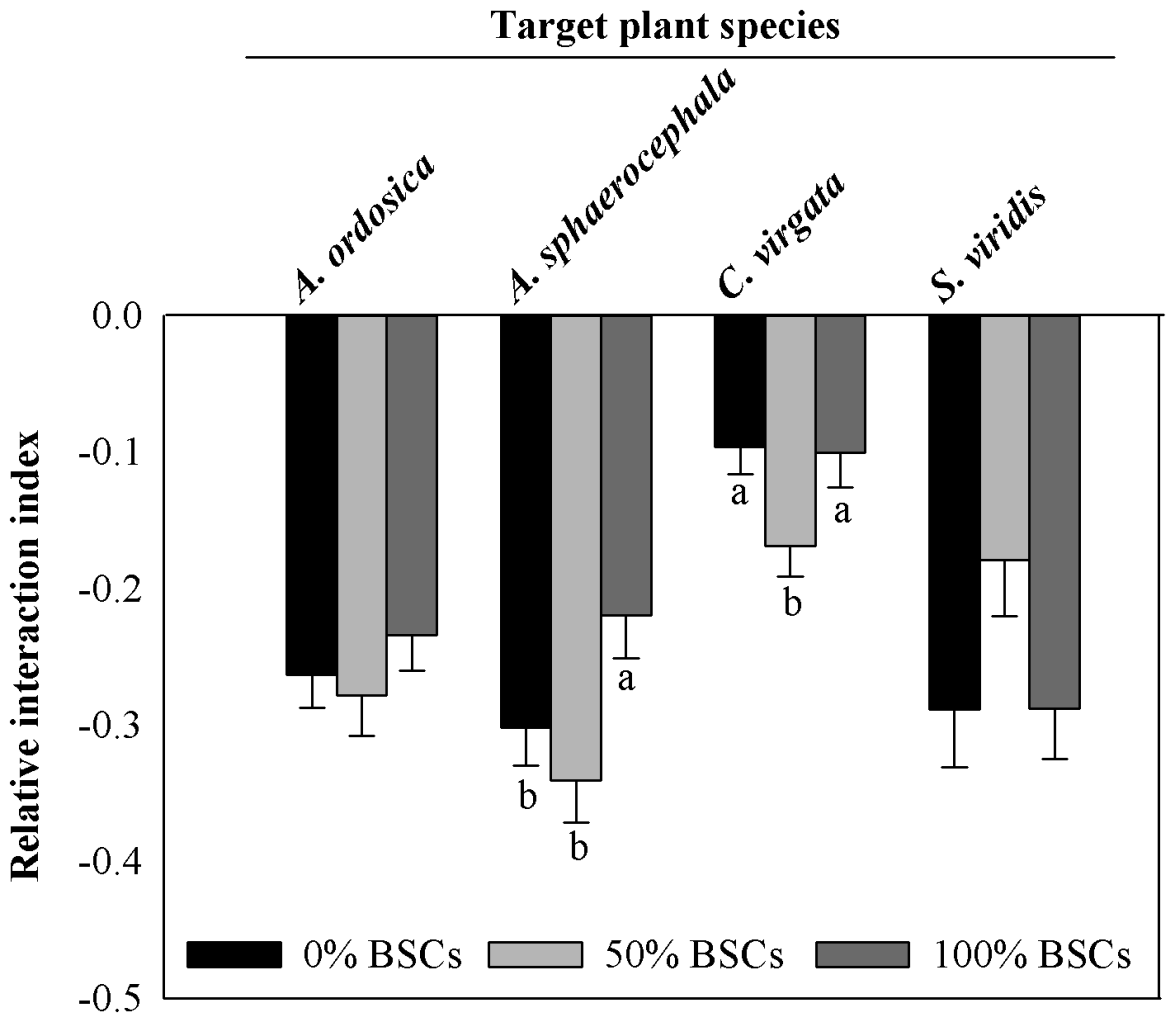
Relative interaction index (RII) (mean ± SE, n = 84) of *Artemisia ordosica*, *Artemisia sphaerocephala*, *Chloris virgata* and *Setaria viridis* in different treatments of biological soil crusts (BSCs) coverage. The plant interaction is (positive) facilitative as RII>0, (negative) competitive as RII<0 and neutral as RII = 0. A greater absolute value of RII indicates a greater intensity of plant interaction. Different letters indicate significant difference at *P*<0.05.

The percent coverage of BSCs had a significant effect on RII value of three target species, *A. sphaerocephala*, *C. virgata* and *S. viridis*, but not for *A. ordosica* (Table 1). BSCs coverage did not change the direction of plant interactions between the four plant species studied here; however, RII was significantly influenced by coverage of BSCs for *A. sphaerocephala* and *C. virgata* (Figure 1). A coverage of BSCs of 100% significantly increased the RII value for *A. sphaerocephala* as well for *C. virgata* (Figure 1). Also, the RII value of *C. virgata* was the lowest with 50% coverage of BSCs (Figure 1).

**Table 1:** *F*-values of three-way ANOVA for effects of water regime (WR), percent coverage of biological soil crusts (BSCs), neighboring plants (NP) and their interactions on the relative interaction index (RII) of the target species of *Artemisia ordosica*, *Artemisia sphaerocephala*, *Chloris virgata* and *Setaria viridis*.

Effects	*A. ordosica*		*A. sphaerocephala*		*C. virgata*		*S. viridis*	
	*F*	*P*	*F*	*P*	*F*	*P*	*F*	*P*
WR	1.545	0.216	16.809	**<0.001**	14.417	**<0.001**	2.009	0.139
BSCs	0.849	0.430	8.931	**<0.001**	7.183	**<0.001**	5.242	**0.007**
NP	47.051	**<0.001**	55.538	**<0.001**	69.595	**<0.001**	36.697	**<0.001**
WR * BSCs	1.318	0.265	5.467	**<0.001**	9.608	**<0.001**	6.925	**<0.001**
WR * NP	2.657	**0.017**	2.780	**0.013**	1.182	0.319	2.396	**0.032**
BSCs * NP	2.185	**0.047**	2.630	**0.019**	1.641	0.139	0.862	0.525
WR * BSCs * NP	1.655	0.081	1.777	0.056	0.759	0.692	3.700	**<0.001**

*P*<0.05 is shown in boldface as significant.

### Interactive effect between BSCs coverage and water regime on plant interactions

**Figure 2:**
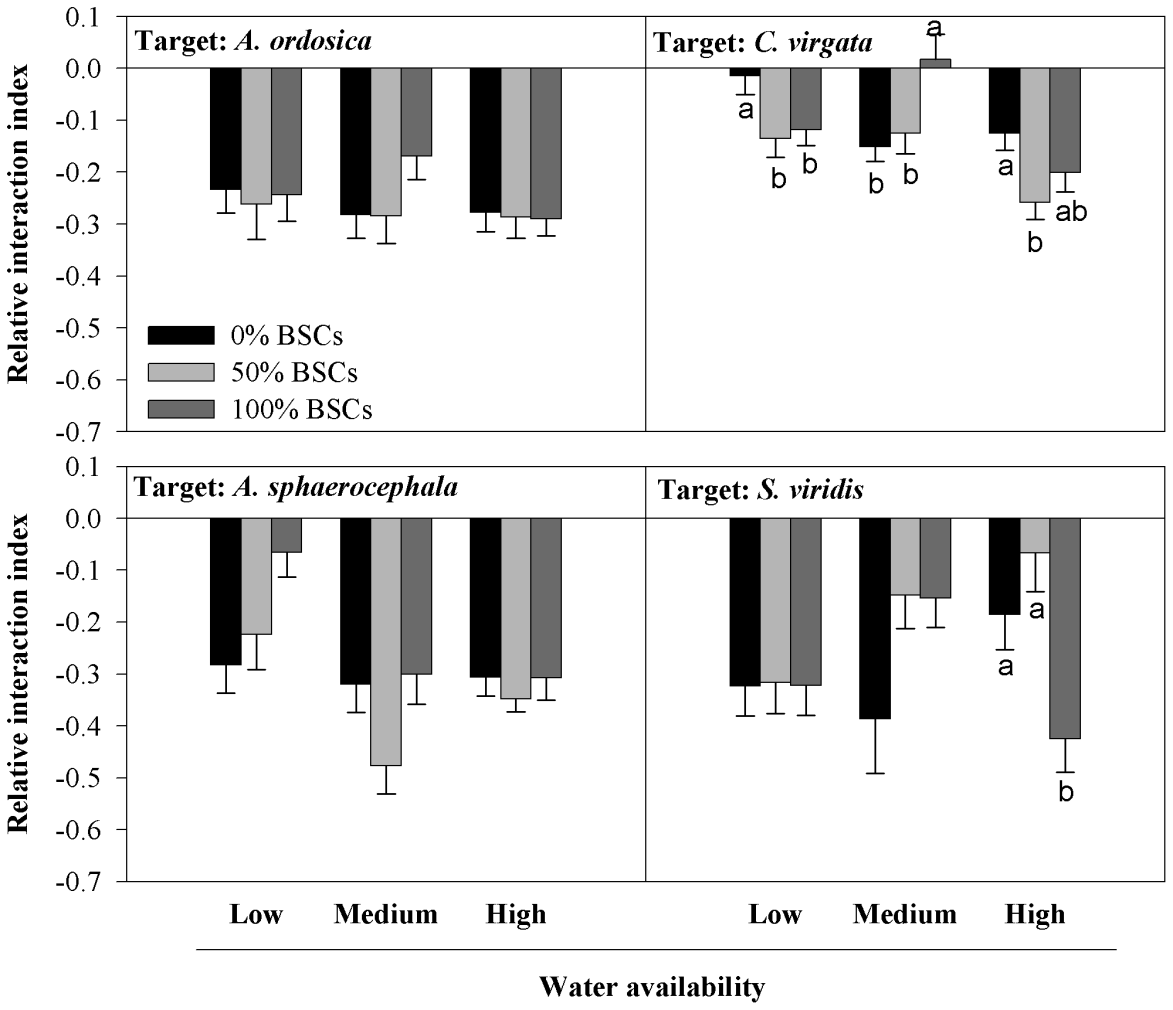
Relative interaction index (RII) (mean ± SE, n = 28) of *Artemisia ordosica*, *Artemisia sphaerocephala*, *Chloris virgata* and *Setaria viridis* in different treatments of simulated rainfall and biological soil crusts (BSCs) coverage. The plant interaction is (positive) facilitative as RII>0, (negative) competitive as RII<0 and neutral as RII = 0. A greater absolute value of RII indicates a greater intensity of plant interaction. Different letters indicate significant difference at *P*<0.05.

Water regime and BSCs coverage had a significant interactive effect on the RII value for *A. sphaerocephala*, *C. virgata* and *S. viridis* (Table 1). Under low and high simulated rainfall conditions, BSCs coverage decreased the RII value for *C. virgata*, while it significantly increased the RII value under medium simulated rainfall condition (Figure 2); BSCs coverage also decreased the RII value for *S. viridis* under high simulated rainfall condition, but no significant effect was observed under low and medium simulated rainfall conditions. Furthermore, under medium simulated rainfall condition, neighbor plant had a facilitative effect for *C. virgata* when BSCs coverage was 100% (RII>0) (Figure 2).

### Plant inter- and intra-specific interactions

**Figure 3:**
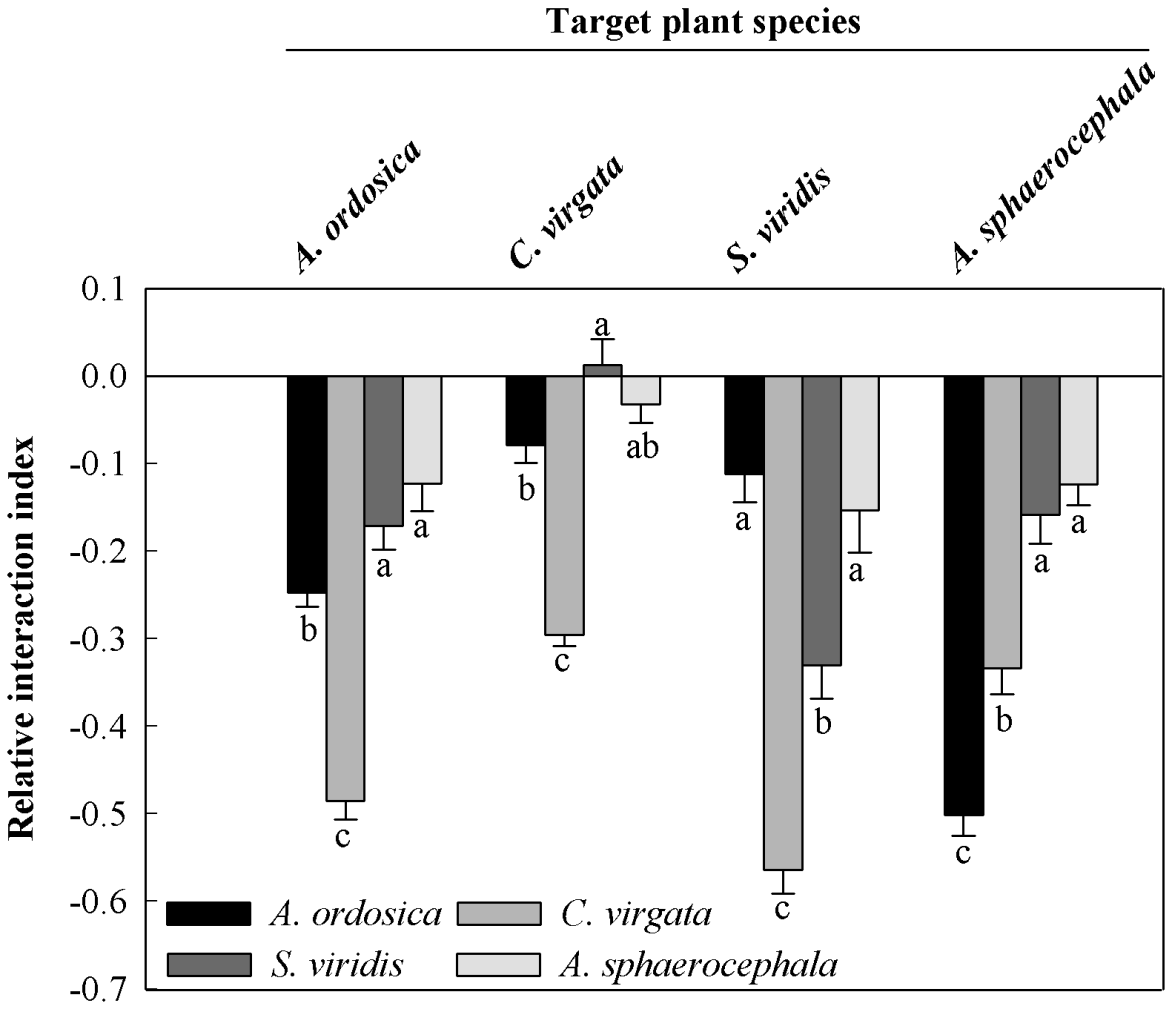
Relative interaction index (RII) (mean ± SE, n = 63) of *Artemisia ordosica*, *Artemisia sphaerocephala*, *Chloris virgata* and *Setaria viridis* with different neighboring plant species. The plant interaction is (positive) facilitative as RII>0, (negative) competitive as RII<0 and neutral as RII = 0. A greater absolute value of RII indicates a greater intensity of plant interaction. Different letters indicate significant difference at *P*<0.05.

For all the pairwise combinations of the four plant species studied here, neighbor plant species had significant influences on the RII value (Table 1), while they had no effect on plant interaction direction except for inter-specific interaction between plants of *C. virgata* and the neighbor plant *S. viridis* (Figure 3). As a neighbor species, *A. sphaerocephala* had a minor effect on all four target plant species as indicated by a higher RII value, while *C. virgata* intensified the competitive effect on the four target species except for *A. sphaerocephala* with the lowest RII value (Figure 3).

### Effect of BSCs coverage on plant biomass

**Figure 4:**
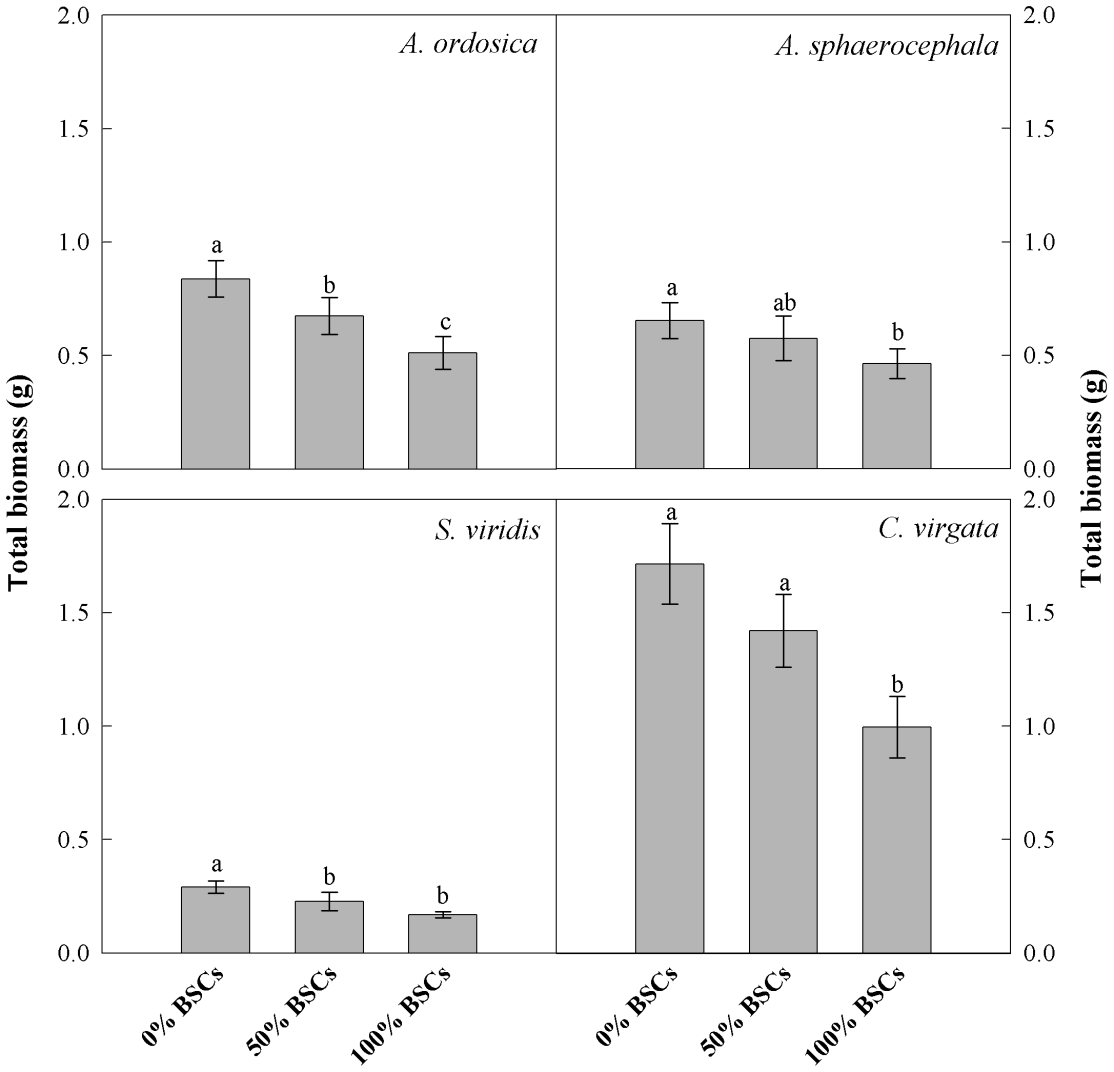
Total biomass (mean ± SE, n = 21) of *Artemisia ordosica*, *Artemisia sphaerocephala*, *Setaria viridis* and *Chloris virgata* in different biological soil crusts (BSCs) treatments. Different letters indicate significant difference at *P*<0.05.

Coverage of BSCs significantly reduced total biomass in all four plant species grown without any neighbor plant (Figure 4). Biomass of *S. viridis* was smallest; while *C. virgata* had the highest biomass (Figure 4).

## Discussion

BSCs coverage can play an important role in affecting individual plants at different stages of their life history, including seed germination [31], [32], seedling survival and establishment [33], [34] and plant growth [35]. Our results showed that percent BSCs coverage had a strong effect on plant interactions (Table 1). Percent BSCs coverage in our experiment did not benefit the growth of individual plants (Figure 4); instead it behaved like a stress factor. This might occur because BSCs sealed the soil surface, inhibiting plant root respiration and reducing water availability. Other studies have shown that stress factors can moderate inter-specific competition intensity [36], [37]. This was partially supported by our results. In our experiment, RII of all four target plant species except *A. ordosica* were significantly affected by BSCs coverage (Table 1). High level of BSCs coverage generally reduced inter-specific competition levels for *A. sphaerocephala* while medium level of BSCs coverage had no significant effects (Figure 1). For the other species (*C. virgata*), effects of BSCs were more complex and did not fit well with the prediction that increased stress caused by BSCs would result in inter-specific interactions being more neutral and less negative. For example, inter-specific competition levels were greatest for *C. virgata* plants with medium BSCs coverage relative to high levels and no BSCs coverage (Figure 1).

Results showed that there were significant interactions between water regime and BSCs coverage when the target species were *A. sphaerocephala*, *C. virgata* or *S. viridis* (Table 1), and high level of BSCs coverage shifted the inter-specific competition to facilitation for *C. virgata* under medium simulated rainfall (Figure 2). Some of the results supported our hypothesis that the effects of BSCs coverage on plant interactions can be modified by water regime. Since BSCs coverage can promote water shortages [38] and increased abiotic stress may shift plant-plant interactions from competitive to facilitative [16], [21], we suspect that annual variation in rainfall amount and variation in BSCs coverage have the potential to produce varied plant-plant interactions. BSCs coverage increased the intensity of competition from neighboring plants for *C. virgata* under both the low and high simulated rainfall conditions, but decreased competition under the medium simulated rainfall conditions (Figure 2). Our current study did not investigate the mechanism (such as plant-soil hydrological processes) of the effects of BSCs coverage.

Size-asymmetric competition appears likely in the experimental plantation [39], because target plant species' competitive stress appeared related to the size of the neighboring plants in our experiment. Neighboring plant species significantly influenced the neighboring plant's competitive intensity or target plant species' competitive stress, and plant interaction was species-specific (Figure 3). When the neighboring plant species was *S. viridis*, which had the smallest plant size due to its low rate of biomass accumulation during the experiment (Figure 4), all four target plant species had lower competitive stress (Figure 3). In contrast, pots with *C. virgata* consistently had negative effects on all target plant species (Figure 3). This strong competitive effect is likely the result of *C. virgata* having the largest plant size of species tested here (Figure 4). This result was consistent with previous research [40], [41], and it fits with the belief that competition can be scaled to the grams of the competitor.

In conclusion, percent coverage of BSCs often had significant effect on plant interactions in our experiment. Also, this effect was species-specific and could be modified by simulated rainfall conditions. Further studies are needed to focus on plant-soil hydrological processes to show how BSCs coverage works ecologically.

## References

[1] Brooker RW (2006) Plant-plant interactions and environmental change. New Phytol 171: 271–284. 16866935 10.1111/j.1469-8137.2006.01752.x

[2] Callaway RM (1995) Positive interactions among plants. Bot Rev 61: 306–349.

[3] Bertness MD, Callaway R (1994) Positive interactions in communities. Trends Ecol Evol 9: 191–193. 21236818 10.1016/0169-5347(94)90088-4

[4] Lehman CL, Tilman D (2000) Biodiversity, stability, and productivity in competitive communities. Am Nat 156: 534–552. 10.1086/303402 29587515

[5] Tilman D, Lehman CL, Thomson KT (1997) Plant diversity and ecosystem productivity: Theoretical considerations. P Natl Acad Sci USA 94: 1857–1861. 10.1073/pnas.94.5.1857 PMC20007 11038606

[6] Connell JH, Slatyer RO (1977) Mechanisms of succession in natural communities and their role in community stability and organization. Am Nat 111: 1119–1144.

[7] Vanandel J, Bakker JP, Grootjans AP (1993) Mechanisms of vegetation succession: a review of concepts and perspectives. Acta Bot Neerl 42: 413–433.

[8] Holzapfel C, Mahall BE (1999) Bidirectional facilitation and interference between shrubs and annuals in the Mojave Desert. Ecology 80: 1747–1761.

[9] Roughgarden J, Diamond J (1986) The role of species interactions in community ecology. In: Diamond J, Case TJ, editors. Community ecology. NY, US: Harper & Row Publishera Inc. pp. 333–343.

[10] McCluney KE, Belnap J, Collins SL, Gonzalez AL, Hagen EM, et al (2012) Shifting species interactions in terrestrial dryland ecosystems under altered water availability and climate change. Biol Rev 87: 563–582. 22098619 10.1111/j.1469-185X.2011.00209.x

[11] Holmgren M, Scheffer M, Huston MA (1997) The interplay of facilitation and competition in plant communities. Ecology 78: 1966–1975.

[12] Choler P, Michalet R, Callaway RM (2001) Facilitation and competition on gradients in alpine plant communities. Ecology 82: 3295–3308.

[13] Armas C, Pugnaire FI (2005) Plant interactions govern population dynamics in a semi-arid plant community. J Ecol 93: 978–989.

[14] Loik ME, Breshears DD, Lauenroth WK, Belnap J (2004) A multi-scale perspective of water pulses in dryland ecosystems: climatology and ecohydrology of the western USA. Oecologia 141: 269–281. 15138879 10.1007/s00442-004-1570-y

[15] Rodriguez-Iturbe I, D'Odorico P, Porporato A, Ridolfi L (1999) On the spatial and temporal links between vegetation, climate, and soil moisture. Water Resour Res 35: 3709–3722.

[16] Schwinning S, Sala OE (2004) Hierarchy of responses to resource pulses in arid and semi-arid ecosystems. Oecologia 141: 211–220. 15034778 10.1007/s00442-004-1520-8

[17] Sher AA, Goldberg DE, Novoplansky A (2004) The effect of mean and variance in resource supply on survival of annuals from Mediterranean and desert environments. Oecologia 141: 353–362. 14669004 10.1007/s00442-003-1435-9

[18] Bowker MA (2007) Biological soil crust rehabilitation in theory and practice: An underexploited opportunity. Restor Ecol 15: 13–23.

[19] Dettweiler-Robinson E, Bakker JD, Grace JB (2013) Controls of biological soil crust cover and composition shift with succession in sagebrush shrub-steppe. J Arid Environ 94: 96–104.

[20] Belnap J (2006) The potential roles of biological soil crusts in dryland hydrologic cycles. Hydrol Process 20: 3159–3178.

[21] Belnap J, Welter JR, Grimm NB, Barger N, Ludwig JA (2005) Linkages between microbial and hydrologic processes in arid and semiarid watersheds. Ecology 86: 298–307.

[22] Ehleringer JR, Schwinning S, Gebauer R (1999) Water use in arid land ecosystems. In: Press MC, Scholes JD, Barker MG, editors. Physiological plant ecology. Oxford: Blackwell Science. pp. 347–365.

[23] Maestre FT, Bowker MA, Canton Y, Castillo-Monroy AP, Cortina J, et al (2011) Ecology and functional roles of biological soil crusts in semi-arid ecosystems of Spain. J Arid Environ 75: 1282–1291. 25908884 10.1016/j.jaridenv.2010.12.008 PMC4404999

[24] Armas C, Ordiales R, Pugnaire FI (2004) Measuring plant interactions: A new comparative index. Ecology 85: 2682–2686.

[25] Cui Y, Lv YZ, Li BG (2004) Physico-chemical properties of soil microbiotic crusts on Erdos plateau. Soils 36: 197–202.

[26] Li XR (2001) Study on shrub community diversity of Ordos Plateau, Inner Mongolia, Northern China. J Arid Environ 47: 271–279.

[27] Zhang XS (1994) Principles and optimal models for development of Maowusu sandy grassland. Acta Phytoecol Sin 18: 1–16.

[28] Li SL, Yu FH, Werger MJA, Dong M, Zuidema PA (2011) Habitat-specific demography across dune fixation stages in a semi-arid sandland: understanding the expansion, stabilization and decline of a dominant shrub. J Ecol 99: 610–620.

[29] Yang XJ, Baskin CC, Baskin JM, Liu GZ, Huang ZY (2012) Seed mucilage improves seedling emergence of a sand desert shrub. Plos One 7: e34597. 22511952 10.1371/journal.pone.0034597 PMC3325279

[30] Oksanen L, Sammul M, Magi M (2006) On the indices of plant-plant competition and their pitfalls. Oikos 112: 149–155.

[31] Deines L, Rosentreter R, Eldridge DJ, Serpe MD (2007) Germination and seedling establishment of two annual grasses on lichen-dominated biological soil crusts. Plant Soil 295: 23–35.

[32] Huang ZY, Gutterman Y (1998) *Artemisia monospermaachene* germination in sand: effects of sand depth, sand/water content, cyanobacterial sand crust and temperature. J Arid Environ 38: 27–43.

[33] Langhans TM, Storm C, Schwabe A (2009) Biological soil crusts and their microenvironment: impact on emergence, survival and establishment of seedlings. Flora 204: 157–168.

[34] Li XR, Jia XH, Long LQ, Zerbe S (2005) Effects of biological soil crusts on seed bank, germination and establishment of two annual plant species in the Tengger Desert (N China). Plant Soil 277: 375–385.

[35] Pendleton RL, Pendleton BK, Howard GL, Warren SD (2003) Growth and nutrient content of herbaceous seedlings associated with biological soil crusts. Arid Land Res Manag 17: 271–281.

[36] Pugnaire FI, Haase P, Puigdefabregas J (1996) Facilitation between higher plant species in a semiarid environment. Ecology 77: 1420–1426.

[37] Graff P, Aguiar MR, Chaneton EJ (2007) Shifts in positive and negative plant interactions along a grazing intensity gradient. Ecology 88: 188–199. 17489467 10.1890/0012-9658(2007)88[188:sipanp]2.0.co;2

[38] Gao SQ, Ye XH, Chu Y, Dong M (2010) Effects of biological soil crusts on profile distribution of soil water, organic carbon and total nitrogen in Mu Us Sandland, China. J Plant Ecol-Uk 3: 279–284.

[39] Potvin C, Dutilleul P (2009) Neighborhood effects and size-asymmetric competition in a tree plantation varying in diversity. Ecology 90: 321–327. 19323214 10.1890/08-0353.1

[40] Lortie CJ, Turkington R (2008) Species-specific positive effects in an annual plant community. Oikos 117: 1511–1521.

[41] Weigelt A, Steinlein T, Beyschlag W (2002) Does plant competition intensity rather depend on biomass or on species identity? Basic Appl Ecol 3: 85–94.

